# Implementing One Health approaches to confront emerging and re-emerging zoonotic disease threats: lessons from PREDICT

**DOI:** 10.1186/s42522-019-0007-9

**Published:** 2020-01-10

**Authors:** Terra R. Kelly, Catherine Machalaba, William B. Karesh, Paulina Zielinska Crook, Kirsten Gilardi, Julius Nziza, Marcela M. Uhart, Erika Alandia Robles, Karen Saylors, Damien O. Joly, Corina Monagin, Prime Mulembakani Mangombo, Placide Mbala Kingebeni, Rudovick Kazwala, David Wolking, Woutrina Smith, PREDICT Consortium, Jonna A. K. Mazet

**Affiliations:** 1 One Health Institute, University of California, Davis, CA USA; 2 EcoHealth Alliance, New York, NY USA; 3 Gorilla Doctors, Mountain Gorilla Veterinary Project and Karen C. Drayer Wildlife Health Center, University of California, Davis, CA USA; 4 Bolivian Network of Primatology, La Paz, Bolivia; 5 Metabiota, San Francisco, CA USA; 6 Sokoine University of Agriculture, Morogoro, Tanzania

**Keywords:** Emerging infectious diseases, Global health, One Health, Zoonotic diseases

## Abstract

Recurring outbreaks of emerging and re-emerging zoonoses, such as Ebola virus disease, avian influenza, and Nipah virus, serve as a reminder that the health of humans, animals, and the environment are interconnected and that early response to emerging zoonotic pathogens requires a coordinated, interdisciplinary, cross-sectoral approach. As our world becomes increasingly connected, emerging diseases pose a greater threat, requiring coordination at local, regional, and global levels. One Health is a multisectoral, transdisciplinary, and collaborative approach promoted to more effectively address these complex health threats. Despite strong advocacy for One Health, challenges for practical implementation remain. Here we discuss the value of the One Health approach for addressing global health challenges. We also share strategies applied to achieve successful outcomes through the USAID Emerging Pandemic Threats Program PREDICT project, which serve as useful case studies for implementing One Health approaches. Lastly, we explore methods for promoting more formal One Health implementation to capitalize on the added value of shared knowledge and leveraged resources.

## Background

Zoonoses lead to millions of deaths annually; the economic losses from a single outbreak can amount to billions of dollars [1, 2]. Recurring outbreaks of emerging and re-emerging zoonotic infectious diseases, such as Ebola virus disease (EVD), severe acute respiratory syndrome (SARS), avian influenza (e.g. H5N1, H7N9), and Nipah virus disease underscore the need to consider the interconnections among the health of humans, animals, and the environment in disease prevention and control measures. As trade and travel facilitate greater access and connections across the world, these zoonoses pose significant and growing global health threats.

Lessons learned from these disease outbreaks highlight the need to shift to a more integrated, holistic, and proactive paradigm, such as can be achieved using the One Health approach. One Health considers the linkages among the health of humans, animals, plants, and their shared environment. As such, the approach allows for a deeper understanding and ability to address the complex eco-social determinants of health and to more effectively and efficiently tackle threats through coordination across disciplines and sectors. One Health approaches are increasingly recognized for their value in addressing emerging infectious disease (EID) threats, as the majority of EIDs arise from wild animal reservoirs in biodiverse landscapes experiencing strong anthropogenic pressures, including human population growth, land use change, and natural resource extraction [3].

At a global level, there is broad support for the concept, which has led to the establishment of several One Health initiatives around the world, including designated divisions within U.S. federal agencies (e.g., in the U.S. the National Park Service One Health Initiative, Centers for Disease Control and Prevention One Health Office, and U.S. Department of Agriculture One Health Coordination Center), interagency working groups and national multisectoral coordination mechanisms (such as Bangladesh’s One Health Secretariat and Liberia’s One Health Coordination Platform) [4–6], international One Health networks and consortia (e.g., the FAO/OIE/WHO Tripartite collaboration, One Health Workforce, One Health Alliance of South Asia, Southeast Asia One Health University Network, One Health Central and Eastern Africa) and One Health designated degree and training programs [7–12]. Furthermore, nearly 50 countries have signed on to the Global Health Security Agenda (GHSA), which was launched in 2014 to bring countries together to promote One Health approaches and strengthen capacities to prevent, detect, and respond to disease threats [13].

Despite this broad support, implementing One Health approaches in practice still proves challenging. For instance, most countries lack formal mechanisms for coordination and integration of activities across the human health, agricultural, and environmental sectors, which are traditionally based in separate ministries or government agencies with different mandates on activities and spending [4, 14]. As a result, practical applications of One Health approaches have largely been ad-hoc [4, 15], resulting in delayed or incomplete prevention and control measures. There is also a need for formal standardized analyses showing the added benefits of One Health over conventional approaches in disease prevention and control [14, 16]. A growing body of research, including studies revealing the financial benefits of One Health investments in addressing emerging zoonoses, is building the evidence base for One Health [17, 18]. However, additional case studies and formal assessments demonstrating the social, health, and economic benefits are needed to garner broader high-level support by decision makers.

In 2009, the US Agency for International Development (USAID) launched the Emerging Pandemic Threats (EPT) Program’s PREDICT Project. PREDICT utilizes a One Health approach focused on early detection and response to potentially zoonotic viral threats at their source ideally before they emerge in people [19]. PREDICT’s efforts have focused on strengthening zoonotic virus surveillance and laboratory capacity in “hotspots” for EIDs. The project provided a platform for breaking down barriers through development of cross-sectoral surveillance and laboratory networks with open sharing of data, coordination on disease outbreak response, and contributions to extant or new national One Health platforms. PREDICT’s efforts to operationalize One Health in collaboration with government and university partners provide valuable examples and evidence for the importance of One Health approaches in addressing complex health challenges. Here we discuss the value of One Health for addressing complex health threats at the human-animal-environment interface and current hurdles for implementing One Health. We also share approaches used by PREDICT to achieve successful outcomes, which serve as useful case studies for applying the One Health approach.

## Value of the One Health approach

The One Health approach builds on existing capacities but is novel in bringing disciplines and sectors together to provide broader health benefits. Increasing cross-sectoral coordination can help promote science-based decision making; reduce unnecessary duplication among the sectors responsible for the health of humans, animals, and the environment; and more effectively address outside factors influencing disease burdens [2, 18].

Comparative medicine has long been acknowledged for its benefits in scientific research, and One Health expands comparative medicine’s scope to surveillance in animals and the environment for early detection and better understanding of threats to mitigate risk and impacts. For example, great ape die-offs associated with Ebola virus have often been detected prior to outbreaks in humans, providing a potential predictive value that can help prevent human cases if paired with risk mitigation measures, such as hunter avoidance of carcasses [20]. Weather conditions have also been used to forecast Rift Valley fever and other outbreaks and can inform vaccination and mosquito control campaigns to reduce health and economic consequences of disease epidemics [21]. Integrated human, animal, and environmental surveillance can likewise elucidate pathways of pathogen sharing and inform development of more comprehensive solutions that emphasize prevention at the source.

The onset of encephalitis cases in people and birds that were ultimately linked to the emergence of West Nile virus in the U.S. in 1999 left public health authorities challenged with identifying its origin. Critical insight into the cause of disease was gained from the veterinary community investigating associated wild bird mortalities. Currently, sentinel surveillance in mosquitos, birds, and horses is used routinely to monitor risk to human health and trigger preventive measures. Parts of North America and Western Europe have also made a concerted effort to control rabies using a One Health approach. While effective rabies control efforts have required substantial investments, they have yielded high public health benefits, with canine vaccination widely considered the most cost-effective strategy [22–24]. The successful control of rabies in dogs through vaccination has then allowed for a targeted approach to managing wildlife reservoirs. Baseline surveillance data has enabled managers to monitor risk and target control efforts in these populations, as seen in response to the rise of raccoon rabies.

An economic optimization projection suggested that investing in a One Health approach through mitigation of pandemic threats versus business-as-usual adaptation could yield a savings of over $300 billion globally over the next century [17]. Similarly, a World Bank analysis suggested that upfront investments of $3.4 billion per year globally in One Health capacity through improved veterinary and public health services could avoid over $30 billion in zoonotic disease response annually worldwide [2].

While these scenarios reflect value for global public good, countries are also increasingly endorsing health security as a national priority given the potential for rapid disease spread via trade and travel networks. This necessitates improved prevention and control of both endemic and emerging disease risks within and beyond a nation’s borders. Climate and other ecological changes are resulting in shifts in geographic ranges of species and their pathogens with a wide range of associated ongoing and novel health threats – ranging from vector-borne and zoonotic diseases to impacts on food safety and security. For example, the spread of Zika virus and CDC’s request to the U.S. government for $1.8 billion to respond demonstrate the need for One Health approaches to implement preventive measures prior to the emergence of novel health threats.

## Case studies: One Health contributions toward more efficient and effective response to emerging zoonotic disease threats

Over the past decade, PREDICT partnered with foreign governments, universities, and other organizations to advance One Health initiatives [19]. In collaboration with local partners, the PREDICT project strengthened capacity for viral surveillance at high-risk animal-human interfaces. Also, when requested by host country government partners, PREDICT provided support during disease outbreaks by incorporating animal sampling into investigations, expanding laboratory analyses to look for novel viruses, and promoting the growth of a trained One Health workforce.

### Rapid outbreak response and containment

During the widespread EVD outbreak in West Africa in 2014, the Democratic Republic of Congo (DRC) experienced its own separate and unique Ebola virus disease outbreak. Unlike West Africa, DRC has a long history of Ebola outbreaks and substantial capacity for response, due in part to a long-running partnership between l’Institut National de Recherche Biomédicale (INRB), the national infectious disease reference laboratory, and other partners like PREDICT. Many experts from the Viral Hemorrhagic Fever Unit of the INRB were deployed in West Africa when the outbreak in DRC occurred. As a result, PREDICT was requested to support laboratory testing. Suspect cases were sampled, specimens were shipped to the PREDICT laboratory at INRB for analyses, and Ebola virus was detected within 1 day of receiving the specimens. Importantly, the strain of Ebola virus detected was distinct from the strain causing the West Africa epidemic, ruling out linkages between the two outbreaks. Following the prompt testing and pathogen identification, the DRC government was able to access the affected area and respond rapidly with contact tracing, dispatching a mobile laboratory, and quarantining suspected cases, leading to swift containment with only 66 cases reported over the two-month duration of this outbreak.

The PREDICT team was also able to assist with collection of wildlife samples from the outbreak area. Contact tracing later identified the likely source of the outbreak as an infected wild animal that had been found dead and butchered for food. This information was key to identifying high-risk practices to target for disease prevention. The rapid response and field investigations informing on prevention measures illustrate what is achievable when an in-country One Health workforce is trained, employed, and ready to act. Such prevention arguably becomes even more important when country capacity to rapidly respond to outbreaks is lacking, especially in fragile areas of high vulnerability to both disease threats and their impacts (e.g. resulting from weak governance structures). The impacts of the on-going EVD outbreak in DRC, which began in Kivu DRC in August 2018, highlight the challenge of responding to a disease outbreak in a remote location where access and control efforts have been substantially impeded by violence and insurgency. These reinforce the need for continued capacity strengthening and integration of sectors at national and sub-national levels, tailored to the local risk context and stakeholders to promote relevance, sustainability, and ownership.

### Prevention of human disease outbreaks

Currently, response to outbreaks around the world is highly reactive, with control measures employed once an outbreak in humans has been detected. PREDICT activities in Bolivia demonstrated that monitoring for zoonotic viruses in wild animals can be a valuable early detection tool for preventing disease outbreaks, particularly in landscapes undergoing substantial alteration, such as deforestation, where breakdown of natural barriers leads to increased contact between wildlife and people.

Yellow fever (YF) is a zoonotic viral hemorrhagic disease [25] that is perpetuated in a transmission cycle involving mosquitos and non-human primate hosts. Because New World primate hosts are especially susceptible to YFV infection, acute clusters of mortality in these populations can signal YFV activity and alert authorities to increased risk of human infection, thereby serving as an early warning system.

In 2012, staff at a wildlife sanctuary in Bolivia, who had received training in wildlife disease surveillance through PREDICT, discovered six dead howler monkeys (*Alouatta sara*) near the park. In collaboration with the sanctuary, PREDICT investigated the mortality event. Post-mortem examinations and diagnostic testing performed at the University of San Andres’ Institute of Molecular Biology and Biotechnology, PREDICT’s partner laboratory in Bolivia, indicated infection by a flavivirus, the family of viruses to which YFV belongs. PREDICT partners reported the results to the Ministry of Health, while conducting further laboratory analyses to confirm that infection was caused by YFV. The Ministry of Health, Pan-American Health Organization, and PREDICT conducted a joint risk assessment followed by a prompt cross-sectoral, coordinated response in the affected area. The response included preventive YF human vaccination, public education and outreach, and mosquito control to reduce risk of infection.

Although YF outbreaks had never been documented in Bolivian primates, authorities were able to implement preventive measures in the surrounding area within 1 week of detection of the mortality event. No human cases of YF were subsequently reported, suggesting the value of early warning systems for increased zoonotic disease risk, local pathogen detection capacity, effective collaboration channels across sectors, and prompt implementation of public health measures for preventing pathogen spillover from animals into people.

### Systematic coordinated data sharing and national One Health platforms

PREDICT worked with foreign government partners to establish a systematic One Health approach to communicating findings stemming from disease surveillance. The process involved sharing laboratory results with designated points of contact in the ministries representing public health, livestock/agriculture, and wildlife, which facilitated discussions on coordinated solutions. It also established open communication channels that enabled more rapid coordinated responses to disease outbreaks. In Rwanda and Tanzania, this collaborative approach was the impetus for PREDICT’s involvement in the development of national One Health platforms in the countries.

In Rwanda, PREDICT-trained personnel served on the Government of Rwanda’s One Health Steering Committee. The committee, which is made up by representatives from the animal and human health and environmental sectors, applied “a participatory and consensus building process” to develop an integrative framework for solving problems at the animal-human-environmental interface [26]. As part of the committee, PREDICT team members aided in the development of a One Health Strategic Plan in 2015 [26]. The plan references commitments to enhance cross-sectoral collaboration and increase One Health workforce capacity in Rwanda. It outlines an implementation strategy covering organizational structure and pooling and mobilizing resources [27]. The Steering Committee oversees the plan, including prioritization of resource allocations, and coordinates the technical aspects of the strategy, which are integrated into the annual action plans of the implementing partners. If successfully operationalized, Rwanda’s One Health Strategic Plan will lead to more efficient and timely responses to disease threats [27].

For example, following the avian influenza (AI) outbreak in neighboring Uganda in 2017, the Rwanda Agriculture Board, in collaboration with representatives from the National One Health Steering Committee, conducted a field investigation of an avian mortality event in Rwanda. In the process of their investigation, they conducted public sensitization around AI risk through informal community meetings and radio broadcast. Although AI was not confirmed in Rwanda, the collaborative efforts initiated by the committee raised critical awareness and led to improvements in Rwanda’s National Contingency Plan against AI highlighting the benefits of this plan to improving preparedness.

Alongside Rwanda, Tanzania also launched its One Health Strategic Plan in 2015. This plan laid the groundwork for multi-sectoral coordination and established a One Health Coordination Unit overseen by a One Health Steering Committee, comprised of secretaries of participating ministries and supported by five technical working groups. Tanzania was the first country to undergo a self-assessment using the World Health Organization (WHO) Joint External Evaluation (JEE) tool, which is a voluntary, collaborative process to assess a country’s capacity to prevent, detect, and rapidly respond to public health threats [28]. PREDICT representatives served in one of the technical working groups using the tool to evaluate strengths, gaps, and priority actions for enhancing national health security. The assessment was instrumental for encouraging cross-sectoral communication and identifying activities in which ministry partners could work together to combat disease threats. The process paved the way for developing the Tanzania National Action Plan for Health Security, which addresses gaps identified by the evaluation. As a culmination of these efforts, Tanzania formally launched the first national One Health Platform and One Health Strategic Plan in 2018 [29].

## The way forward: implementing One Health

While mechanisms for operationalizing One Health are variable across contexts, case studies demonstrating successful One Health outcomes can provide valuable insight for implementing approaches elsewhere. These can be leveraged as countries work toward multisectoral coordination platforms with more sustainable approaches to One Health (such as through the establishment of the Zoonotic Disease Unit in Kenya [30]). These platforms often have high political will, with oversight and support at prime minister or presidential levels which promote country ownership and sustained attention and across sectors. Over the past 5 years, the GHSA has been instrumental in creating an enabling environment and political will for strengthening global and national health securities through a One Health approach. JEEs conducted in several countries around the world have revealed weaknesses in coordination across health sectors prompting the recommendation to develop national One Health platforms. To work towards this goal, the World Bank, USAID EPT program, and United Nations organization partners have compiled resources to assist countries with formalizing a One Health strategy, including tools for capacity assessments, resource mapping and prioritization, and One Health systems improvement [24, 31–37]. These tools aid in identifying where investments in One Health approaches and leveraged resources could fill gaps, avoid unnecessary overlap, and result in more holistic, preventive approaches [18]. In allocating resources, it is beneficial to conduct formal standardized assessments to evaluate how best to optimize investments to ensure added value gained by integrating efforts across health sectors [32, 33]. For example, One Health approaches have yielded higher returns on investments through joint human-animal disease surveillance and prevention and control measures, including vaccination campaigns [18, 19, 34]. Cross-sectoral exercises to assess risk and economic impacts of zoonoses have also brought stakeholders to the table to facilitate more systematic collaboration and communication and to identify opportunities of mutual benefit [18, 35, 36]. Leveraging the One Health approach to ensure the wider risk context and relevant sectors, especially at sub-national levels, can help boost countries’ abilities to prepare for a suite of current and evolving threats.

Finally, it is critical to continue to raise awareness of One Health and foster leaders who are uniquely skilled to work across disciplines and sectors. Around the world, universities are progressively incorporating One Health education into their curricula, including designated degree programs. These programs need to be developed around a set of core competencies with an emphasis on practical skill-building [37] to provide students with the knowledge and experience necessary to address complex health threats.

## Conclusions

While there is increasing commitment to One Health across the world, implementing One Health approaches in practice still proves challenging. Development of national One Health platforms and policies are critical for improving coordination and integration of activities and programs across sectors. In many countries, the GHSA has provided a platform for coordination and served as the impetus to initiate One Health strategic plans and to develop national One Health policies. In addition, support from international organizations, such as the World Bank, USAID (EPT Program), and UN partners has aided several countries in designing and implementing One Health strategies and in strengthening national One Health systems [18, 19]. While some programmatic activities may not be feasible in the absence of external funding, one route for sustainability is the application of low-cost coordination systems that have been tested and validated, including routine inter-ministry meetings to share disease surveillance results and discuss coordinated mitigation efforts. Country investments in human and animal health systems, including through development loans, illustrate the value that countries place on enhancing capacity for disease preparedness. Further, there is a need to continue to bring attention to the value of One Health approaches and to invest in training a workforce of One Health leaders who have the skills to think critically and work collaboratively across sectors.

## Data Availability

Not applicable.

## Abbreviations

AI: Avian Influenza
CDC: Centers for Disease Control and Prevention
DRC: Democratic Republic of Congo
EID: Emerging Infectious Disease
EPT: Emerging Pandemic Threats
EVD: Ebola Virus Disease
GHSA: Global Health Security Agenda
INRB: l’Institut National de Recherche Biomédicale
JEE: Joint External Evaluation
UN: United Nations
USAID: US Agency for International Development
WHO: World Health Organization
YF: Yellow Fever
